# Exploring the Effects of Personality Traits on the Perception of Emotions From Prosody

**DOI:** 10.3389/fpsyg.2019.00184

**Published:** 2019-02-12

**Authors:** Desire Furnes, Hege Berg, Rachel M. Mitchell, Silke Paulmann

**Affiliations:** ^1^Department of Clinical Psychology, University of East Anglia, Norwich, United Kingdom; ^2^School of Biological Sciences, University of East Anglia, Norwich, United Kingdom; ^3^Centre for Affective Disorders, King's College, London, United Kingdom; ^4^Department of Psychology, Centre for Brain Science, University of Essex, Colchester, United Kingdom

**Keywords:** emotional prosody, personality traits, emotional recognition accuracy, emotional recognition speed, tone of voice, vocal emotion

## Abstract

It has repeatedly been argued that individual differences in personality influence emotion processing, but findings from both the facial and vocal emotion recognition literature are contradictive, suggesting a lack of reliability across studies. To explore this relationship further in a more systematic manner using the Big Five Inventory, we designed two studies employing different research paradigms. Study 1 explored the relationship between personality traits and vocal emotion *recognition accuracy* while Study 2 examined how personality traits relate to vocal emotion *recognition speed*. The combined results did not indicate a pairwise linear relationship between self-reported individual differences in personality and vocal emotion processing, suggesting that the continuously proposed influence of personality characteristics on vocal emotion processing might have been overemphasized previously.

## Introduction

One of the most influential hypotheses examining differences in emotion processing, the trait-congruency hypothesis, argues that stable personality traits influence the precision of an individual's emotion processing (see Rusting, 1998 for review). For example, extraversion and neuroticism have been extensively linked to processing of positive and negative emotions, respectively (Larsen and Ketelaar, 1989; Gomez et al., 2002; Robinson et al., 2007). However, although evidence points toward some form of relationship between selective emotion processing and certain personality characteristics, the literature from recent decades is contradictive (Matsumoto et al., 2000).

Both the *vocal* and *facial* emotion recognition literature has explored the relationship between different personality traits and emotion recognition *accuracy* (although far more emphasis has been put on detecting emotions from faces). For instance, in the vocal emotion literature, extraversion and conscientiousness have been associated with better vocal emotion recognition, but only in males (Burton et al., 2013). In contrast, Terracciano et al. (2003) found a positive relationship between vocal emotion perception and openness to experience. Similarly, in the facial emotion literature, some studies have found a link between better emotion recognition and openness to experience and conscientiousness (Matsumoto et al., 2000). In contrast, other studies have emphasized the importance of extraversion and neuroticism. Confusingly, while some researchers have argued that extraverted individuals perform better on facial emotion recognition tasks (Matsumoto et al., 2000; Scherer and Scherer, 2011), other studies have failed to evidence this relationship (Cunningham, 1977). Similarly, neuroticism has been linked to both *poorer* (Matsumoto et al., 2000) and *better* (Cunningham, 1977) recognition of facial emotions. It is thus evident that the confusing and contradictory relationships between personality traits and emotion recognition are not wholly consistent with predictions made by the trait-congruency hypothesis in either the facial or vocal domains (see Table 1 for an overview).

**Table 1:** An overview of studies exploring the relationship between personality traits and emotion recognition accuracy.

**Expressed emotion**	**Reference**	**Emotion recognition measure**	**Personality inventory**	**Main findings**	**Effect size (r) (BFI variables)**					**N**
					**E**	**A**	**C**	**N**	**O**	
Facial	Rubin et al., 2005	DANVA	BFI	No direct effects found between emo.rec. and personality variables, but moderating effect of **E** between leadership and emo.rec.	0.03^a^ (NS)	0.11^a^ (NS)	–	–	–	145
	Matsumoto et al., 2000 (Study 5)	JACBART (Version 3)	NEOPI-R	Positive relationship between emo.rec. and **O** and **C**.	NS	NS	0.12 (NS) (anger) −0.20 (NS) (contempt) 0.39^*^ (disgust) 0.25 (NS) (fear) 0.01 (NS) (happiness) 0.40^**^ (sadness) 0.21 (NS) (surprise)	NS	0.27 (NS) (anger) −0.07 (NS) (contempt) 0.50^***^ (disgust) 0.23 (NS) (fear) −0.11 (NS) (happiness) 0.29 (NS) (sadness) 0.38^*^ (surprise)	44
	Matsumoto et al., 2000 (Study 5)	JACBART (Version 3)	BFI	Positive relationship between emo.rec. and **O** and **C**.	NS	NS	0.17 (NS) (anger) −0.09 (NS) (contempt) 0.41^**^ (disgust) 0.36^*^ (fear) −0.00 (NS) (happiness) 0.45^**^ (sadness) 0.38^*^ (surprise)	NS	0.20 (NS) (anger) 0.12 (NS) (contempt) 0.38^*^ (disgust) 0.17 (NS) (fear) 0.06 (NS) (happiness) 0.17 (NS) (sadness) 0.30 (NS) (surprise)	44
	Matsumoto et al., 2000 (Study 4)	JACBART (Version 2)	EPI	Positive relationship between **E** and facial emo.rec. Negative relationship between **N** and facial emo.rec.	0.37^*^ (anger) −0.08 (NS) (contempt) 0.61^***^ (disgust) 0.34^*^ (fear) 0.60^***^ (happiness) 0.29 (NS) (sadness) 0.33^*^ (surprise)	–	–	−0.51^**^ (anger)−0.00 (NS) (contempt)−0.35^*^ (disgust)−0.08 (NS) (fear)−0.39^*^ (happiness)−0.51^**^ (sadness)−0.36^*^ (surprise)	–	27
	Elfenbein et al., 2007	Study specific	IPIP-NEO	No relationship between personality variables and facial emo.rec.	0.08^a^ (NS)	–	−0.03^a^ (NS)	0.01^a^ (NS)	0.07^a^ (NS)	164
	Banziger et al., 2009	MERT	NEO-FFI	No relationship between personality variable and facial emo.rec.	NS	–	–	NS	NS	72
	Cunningham, 1977	Study specific	EPI	Positive relationship between **N** and facial emo.perc.	−0.10^a^ (NS)	–	–	0.32^*^^a^	–	36
	Scherer and Scherer, 2011	ERI	CAPP	Positive relationship between **E** and facial emo.rec. Negative relationship between **N** and facial emo.rec.	0.06^**^^a^	–	–	0.01^a^ (NS)	–	72
	Terracciano et al., 2003	PAT (CAU faces)	NEO-FFI: A.A. Sample NEO-PI: CAU Sample	Positive relationship between **O** and facial emo.rec. However, CAU faces used as stimuli in *both* A.A. and CAU samples.	0.10^a^ (NS) (A.A.) 0.03^a^ (NS) (CAU)	0.05^a^ (NS) (A.A.)0.12^a^ (NS) (CAU)	−0.13^a^ (NS) (A.A.) 0.14^a^ (NS) (CAU)	0.06^a^ (NS) (A.A.)−0.17^a^ (NS) (CAU)	0.24^**^^a^ (A.A.) 0.30^*^^a^ (CAU)	106 A.A.46 CAUs
Vocal	Cunningham, 1977	Study specific	EPI	No relationship between **E, N** and vocal emo.perc.	−0.03^a^ (NS)	–	–	0.25^a^ (NS)	–	36
	Terracciano et al., 2003	PAT	NEO-FFI: A.A. sample NEO-PI: CAU sample	Positive relationship between **O** and vocal emo.rec.	0.14^a^ (NS) (A.A.) −0.04^a^ (NS) (CAU)	0.10^a^ (NS) (A.A.)−0.14^a^ (NS) (CAU)	−0.06^a^ (NS) (A.A.) 0.08^a^ (NS) (CAU)	0.01^a^ (NS) (A.A.)−0.03^a^ (NS) (CAU)	0.28^**^^a^ (A.A.) 0.25^^*^a^ (CAUs)	106
	Burton et al., 2013	DANVA2	NEO-FFI	*Negative* association between **E** and **C** and vocal emo.rec. *errors* in males, but not females.	−0.50^*^^a^ (males) 0.06^a^ (NS) (females)	−0.21^a^ (NS) (males)−0.11^a^ (NS) (females)	−0.28^*^^a^ (males) −0.13^a^ (NS) (females)	0.06^a^ (NS) (males)0.11^a^ (NS) (females)	−0.02^a^ (NS) (males) 0.01^a^ (NS) (females)	115 (73 females)
	Scherer and Scherer, 2011	ERI	CAPP	Positive relationship between **E** and vocal emo.perc. Neurotic individuals poorer at recognizing vocal emotions compared to emotionally stable individuals.	0.17^**^^a^	–	–	0.06^**^^a^	–	72
	Banziger et al., 2009	MERT	NEO-FFI	No relationship between personality variables and vocal emo.perc.	NS	–	–	NS	NS	72

DANVA, Diagnostic Analysis of Nonverbal Accuracy; NEO-PI, NEO Personality Inventory; NEO PI-R, Revised NEO Personality Inventory; EPI, Eysenck Personality Inventory; JACBART, Japanese and CAU Brief Affect Recognition Test; ERI, Emotion Recognition Index; PAT, perception of affect task; BFI, Big Five Inventory; MERT, The Multimodal Emotion Recognition Test; CAPP, Computer Assessment of Personal Potential; NEO-FFI, NEO Five Factor Inventory; IPIP-NEO, International Personality Item Pool. A.A., African American; CAU, Caucasian; E, Extraversion; A, Agreeableness; C, Conscientiousness; N, Neuroticism; O, Openess to experience; NS = not significant;

* < 0.05.

** < 0.01.

*** < 0.001.

a *, mean recognition accuracy (i.e., some studies only correlate personality variables with overall recognition accuracy and not for separate emotions); –, not tested*.

One set of factors that might potentially explain the inconsistent findings relate to methodological differences between studies. For instance, different studies have used different personality inventories and varying emotion recognition measures. While some studies correlate personality traits against overall emotion recognition accuracy (Terracciano et al., 2003; Elfenbein et al., 2007; Burton et al., 2013), some studies have investigated the relationship between personality traits and recognition of specific emotions (e.g., Matsumoto et al., 2000). Further, some studies have relied on specific individual personality traits, such as extraversion and neuroticism alone (e.g., Cunningham, 1977; Scherer and Scherer, 2011), whereas other studies have included all Big Five (i.e., agreeableness, conscientiousness, extraversion, neuroticism, and openness to experience) personality dimensions (e.g., Matsumoto et al., 2000; Rubin et al., 2005). It is thus clear that our understanding of the potential dynamic interplay between personality traits and processing of emotional information is far from complete and warrants further investigation.

Continuing from the confusing literature on personality and vocal emotion recognition accuracy, it is similarly possible that individual differences in personality traits may influence the temporal processing of vocal emotions. For instance, while the trait-congruency hypothesis would predict that extraversion and neuroticism are linked to *better recognition* of positive and negative emotions, it could also be argued that extraversion and neuroticism are linked to *quicker recognition* of positive and negative emotions, respectively. Recent advances in the vocal emotion literature have allowed investigation of the temporal processing of vocal emotions, which can provide crucial information on *when* distinct emotion categories are recognized and *how much* acoustic information is needed to recognize the emotional state of a speaker (Pell and Kotz, 2011).

The auditory gating paradigm is often employed when examining how much acoustic-phonetic information is required to accurately identify a spoken stimulus and can be used to examine any linguistic stimulus (e.g., word, syllable, sentence) of interest (Grosjean, 1996). For example, a spoken word can be divided into smaller segments and listeners are then presented with segments of increasing duration starting at stimulus onset. The first segment is thus very brief while the final segment corresponds to the complete stimulus (Grosjean, 1996). After listening to each segment listeners are asked to identify the target word and rate how confident they are in the accuracy of their response. This technique enables calculation of the isolation point, or the size of the segment needed for accurate identification of the target (Grosjean, 1996).

Different emotion categories unfold at different rates (Banse and Scherer, 1996) which can be understood in terms of the biological significance of the emotion category (Pell and Kotz, 2011). For example, fear signals a threatening situation that requires an immediate behavioral response, which suggests that this emotion category should be recognized faster than a less threatening emotion, such as happiness. In line with this, Pell and Kotz (2011) found an emotion bias, in which fear was the quickest recognized emotion category. In contrast, Cornew et al. (2010) have argued for a neutral bias, as they found that neutral utterances were identified more rapidly than angry utterances, which were identified more rapidly than happy utterances. The position of acoustical cues has also shown to play a crucial role in the decoding process of vocal emotions. Rigoulot et al. (2013) explored recognition patterns where the first gate corresponded to the last segment before sentence *offset*, the first gate reflected sentence *onset*, and the final gate corresponded to the full utterance of the sentence backwards. Results revealed that the position of acoustical cues is particularly important when prosodic cues of happiness and disgust are expressed.

While the behavioral literature on the time course processing of vocal emotions is still in its infancy, research on how differences in personality traits influence temporal processing of vocal emotions is absent. To our knowledge, the only study that has examined differences in temporal processing of vocal emotions, although at a group level, is the study by Jiang et al. (2015). They examined the time course of vocal emotions across cultures and reported an in-group advantage, i.e., quicker and more accurate recognition of stimuli, when English and Hindi listeners were presented with emotionally intoned vocal utterances presented in their own language, compared to foreign language utterances (English for Hindi listeners, Hindi for English listeners). This is consistent with findings from the vocal emotion accuracy literature, in which other studies (e.g., Paulmann and Uskul, 2014) also reported an in-group advantage in recognizing emotional displays. However, it is yet unexplored how the temporal dynamics of vocal emotions are influenced by personality characteristics.

The present investigation consisted of two independent but related studies based on two main aims; to get a better understanding of whether and how personality traits can predict individual differences in (1) vocal emotion *recognition accuracy*, and (2) vocal emotion *recognition speed*. Specifically, while Study 1 investigates whether *recognition accuracy* of various vocal emotions (i.e., anger, disgust, fear, happiness, neutral, pleasant surprise, and sad) is related to individual differences in personality traits, Study 2 is the first attempt to explore the influence of individual personality traits on the *time course processing* of vocal emotions. Thus, it asks if individuals differ in the amount of acoustic information they require to draw valid conclusions about the emotion communicated through tone of voice.

The two studies were also designed to address certain methodological issues identified in the previous literature and to account for other potential confounding variables. Firstly, the Big Five Inventory (BFI) was used consistently across both studies to ensure that potential findings were not confounded by the use of different measurement tools. Recognition rates for individual emotion categories, as well as for overall recognition accuracy, were explored in relation to scores on the BFI, to allow a fuller comparison to the previous literature.

Generally, vocal perception studies tend to use professional actors to portray the emotions (e.g., Graham et al., 2001; Banziger and Scherer, 2005; Airas and Alku, 2006; Toivanen et al., 2006), based on the assumption that professional actors are better able to portray unambiguous emotions (Williams and Stevens, 1972). It has however been argued that professional actors may produce exaggerated stereotypical portrayals (e.g., Scherer, 1995; Juslin and Laukka, 2001; Paulmann et al., 2016), which may result in lack of ecological validity (Scherer, 1989). A recent study by Paulmann et al. (2016) reported that, at an acoustical level, untrained speakers could convey vocal emotions similarly to trained speakers, suggesting that the use of untrained speakers might provide a good alternative. Thus, in this investigation we employed materials from *both* untrained speakers (Study 1) and a professionally trained speaker (Study 2). This allows generalizing potential personality trait effects on emotional vocal recognition across different speaker types (professional and non-professional). This approach will also be of use in future studies when deciding what kind of materials might be best suited to explore personality traits in the context of vocal emotions.

In line with the trait-congruency hypothesis, we hypothesized that extraversion would be linked to *better and quicker* recognition of positive vocal emotions, while neuroticism would be linked to *better and quicker* recognition of negative emotions. To specifically explore this hypothesis in both studies, an overall recognition accuracy score was generated for positive (happy, pleasant surprise) and negative (anger, disgust, fear, sadness) emotions, and was then examined in relation to levels of extraversion and neuroticism. Due to the sparse and contradictory findings in the previous literature, predictions are difficult to make for the other Big Five personality traits i.e., agreeableness, conscientiousness, and openness to experience. We would argue that, if there is a true, systematic relationship between personality traits and processing of vocal emotions, this relationship should be evident across both studies.

## Study 1

The overall aim of Study 1 was to explore the relationship between individual differences in personality traits and vocal emotion recognition *accuracy*.

### Methods

#### Participants

Ninety-five [75 females, mean age: 19.5, SD (standard deviation): 3.09] undergraduate Psychology students at the University of Essex participated and gave their informed written consent. They received module credits for their participation. All participants reported normal or corrected to normal hearing and vision. Participants self-reporting experiencing mental disorders were excluded from the analyses, as several studies have shown impaired emotion recognition in clinical populations such as depression (e.g., Leppanen et al., 2004), schizophrenia (e.g., Kohler et al., 2003), and borderline personality disorder (e.g., Unoka et al., 2011).

Consequently, 81 participants (65 females) were included in the final statistical analyses. This sample size was considered sufficient, as G^*^Power3.1 (Faul et al., 2007) yielded an estimated sample size of 84 participants (power = 0.80, alpha = 0.05, and effect size = 0.3; we considered a small to medium effect size to be a conventional estimate based on several studies exploring similar variables—often with smaller sample sizes, see Table 1).

#### Stimuli Selection

Stimuli for Study 1 were taken from a previous inventory (Paulmann et al., 2016). Fifteen semantically neutral sentences (e.g., “The fence was painted brown”) were portrayed by nine (non-professional) female speakers in seven emotional tones (anger, disgust, fear, happiness, neutral, sad, and surprise). For each emotional category, 40 sentences were presented resulting in 280 sentences in total. Emotionality ratings were obtained for these materials in a previous study (Paulmann et al., 2016). All materials were recognized much better than chance would predict. Specifically, arcsine-transformed Hu scores for materials ranged from 0.42 (for happiness) to 0.96(for anger; see Paulmann et al. (2016) for more details on stimuli). The 280 sentences were randomly allocated into seven blocks consisting of 40 sentences. Sentence stimuli are outlined in Appendix A.

#### The Big Five Inventory (BFI)

The BFI (John et al., 1991, 2008) is a 44-item questionnaire assessing the Big Five (A, C, E, N, O) personality characteristics. In contrast to the NEO-PI-R (Costa Jr and McCrae, 1995), the BFI is a shorter version frequently used in research settings that assesses prototypical traits of the Big Five. In addition, the BFI shares high reliability and validity when compared to other Big Five measures, e.g., Trait-Descriptive Adjectives (TDA) (Goldberg, 1992) and NEO-FFI (a shorter 60-item version of the NEO-PI-R) (Costa and McCrae, 1989, 1992).

#### Design

A cross-sectional design was employed. For the correlational analyses, personality traits were used as predictor variables, while the criterion variable was vocal emotion recognition accuracy. For the repeated-measures ANOVA, Emotion was the within-subject variable with seven levels; anger, disgust, fear, happiness, neutral, pleasant surprise, and sadness.

#### Procedure

Participants were seated in front of a computer where they listened to the sentence stimuli. They were informed of the experimental procedure, both by the experimenter and by on-screen instructions. Five practice trials were included to ensure that participants fully understood the task. For each trial, a fixation cross appeared on the center of the screen before sentence onset and remained visible while participants listened to each sentence stimuli. They were asked to indicate which emotion the speaker intended to convey using a forced-choice format, in which seven emotion boxes (anger, disgust, fear, happy, neutral, pleasant surprise, sad) appeared on the screen after sentence offset. After the response was given, there was an inter-stimulus interval of 1,500 ms before the next sentence stimulus was presented. In-between the four blocks, participants were able to pause until they felt ready to continue the task. The total run-time of the computerized task was approximately 30 min. After finishing the experiment, participants completed the BFI, the Satisfaction of Life Scale, PANAS-X and the Affect Intensity Measure (latter three all not reported here) before they were debriefed about the study purpose. All measures and procedures applied are reported within this manuscript.

### Results

#### Vocal Emotion Recognition

To control for stimulus and response biases, raw hit rates were transformed into unbiased hit rates (H_u_ scores; Wagner, 1993) (see Appendix C for raw hit rates and error patterns of responding). As H_u_ scores are proportional scores, they were arcsine-transformed as recommended for these data (Wagner, 1993). The arcsine-transformed H_u_ scores are presented in Table 2; a score of zero is equivalent to chance performance while a score of 1.57 reflects perfect performance.

**Table 2:** Mean arcsine-transformed H_u_ scores and SD for each emotion and averaged across all emotions.

	**Intended emotion**							
	**Anger**	**Disgust**	**Fear**	**Happy**	**Neutral**	**Pls.sur**	**Sad**	**Average**
Recognition accuracy	0.81	0.53	0.54	0.43	0.55	0.73	0.65	0.60
SD	0.13	0.17	0.17	0.15	0.11	0.10	0.11	0.09

*Pls. sur, pleasant surprise*.

To examine whether some emotions are easier to identify than others, a repeated-measures ANOVA was conducted using a modified Bonferroni procedure to correct for multiple comparisons (Keppel, 1991). In this procedure, the modified alpha value is obtained in the following way: alpha multiplied by the degrees of freedom associated with the conditions tested, divided by the number of planned comparisons. The Greenhouse-Geisser correction was applied to all repeated-measures with greater than one degree of freedom in the numerator.

A significant main effect was found for Emotion, *F*_(4.579, 366.301)_ = 104.179, *p* < 0.001, suggesting that some emotions are indeed better recognized than others. *Post hoc* comparisons revealed that all emotion contrasts were significantly different from each other, with the exception of the contrast between disgust and fear, disgust and neutral, and fear and neutral. As can be seen in Table 2, anger was the emotion category recognized most accurately, while happy was the poorest recognized emotion.

#### Vocal Emotion Recognition and Personality

**Figure 1:**
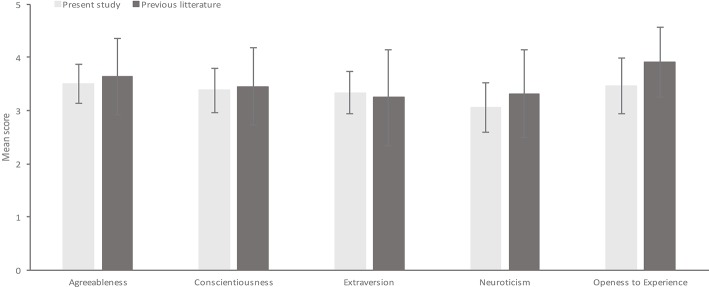
A comparison of means and SDs from the present study for each BFI variable and means and SDs obtained for the same variables in previous research. Means from the previous literature are based on results reported by Srivastava et al. (2003), where a mean age of 20 years was used for comparison, as the mean age of the present sample was 19.5 years.

Means and standard deviations (SDs) were calculated for all the five personality dimensions and compared to the previous literature as compiled by Srivastava et al. (2003). Results from the present study were considered as a valid representation of administration of this measure to a population sample (see Figure 1) though our standard deviations look slightly smaller in some instances.

Pearson's correlations were conducted to examine the relationship between arcsine-transformed H_u_ scores and BFI scores (see Table 3). No significant relationship was found between overall emotion recognition and any of the Big Five personality traits. Similarly, no correlation was evident between extraversion and neuroticism and positive and negative emotion categories, respectively. However, a negative relationship was observed between recognition of positive emotions and openness to experience, *r* = −0.240, *p* = 0.031, showing that individuals scoring lower on openness to experience were better at recognizing positive vocal emotions.

**Table 3:** Study 1: Pearson's correlations (*R*-value) and their significance level (^*^P < 0.05) between H_u_ scores and the Big Five Inventory (BFI).

		**Intended emotion**										
**Measure**		**Anger**	**Disgust**	**Fear**	**Happy**	**Neutral**	**Pls.sur**	**Sad**	**EmoAve**	**AveNotNeu**	**AveNegEm**	**AvePosEm**
Agreeableness	*r*-value	−0.016	0.280^*^	−0.139	0.136	−0.013	0.011	−0.102	0.050	0.058	0.024	0.108
	*p*-value	0.889	0.011	0.217	0.226	0.912	0.921	0.367	0.656	0.609	0.829	0.338
Conscientiousness	*r*-value	−0.087	−0.003	0.030	−0.011	0.221^*^	0.109	0.053	0.054	0.016	−0.002	0.048
	*p*-value	0.438	0.980	0.790	0.919	0.047	0.334	0.635	0.633	0.886	0.989	0.669
Extraversion	*r*-value	−0.046	0.022	−0.118	−0.110	−0.084	−0.157	−0.022	−0.109	−0.104	−0.058	−0.164
	*p*-value	0.685	0.843	0.294	0.328	0.456	0.161	0.849	0.331	0.356	0.607	0.142
Neuroticism	*r*-value	−0.047	−0.014	0.128	−0.086	0.200	0.190	0.051	0.078	0.047	0.045	0.035
	*p*-value	0.675	0.904	0.253	0.444	0.074	0.089	0.653	0.487	0.675	0.689	0.760
Openness to experience	*r*-value	−0.099	−0.137	0.069	−0.303^**^	−0.082	−0.024	−0.231^*^	−0.175	−0.177	−0.117	−0.240^*^
	*p*-value	0.380	0.223	0.542	0.006	0.466	0.829	0.038	0.117	0.115	0.297	0.031

*The table lists correlations between H_u_ scores for each emotion category (Pls.sur, pleasant surprise) and the average for all emotions (EmoAve). It also shows correlations between all emotions except neutral (AveNotNeu: anger, disgust, fear, happy, pls.sur, and sad), all negative emotions (AveNegEm: anger, disgust, fear, and sad) and all positive emotions (AvePosEm: happy and pls.sur)*.

### Discussion

Study 1 aimed to explore whether individual differences in personality traits could predict variation in vocal emotion perception. Group data analyses of emotion perception in isolation replicated findings previously reported in the vocal emotion literature (e.g., see Scherer, 1989 for a review). However, no noteworthy relationship was found between overall vocal emotion perception and any of the five personality dimensions, or between extraversion and neuroticism and positive and negative emotion categories, respectively. The present study thus failed to support the predictions made by the trait-congruency hypotheses. However, it should be noted that previous findings are also only partially in line with the trait-congruency predictions. For instance, Scherer and Scherer (2011) and Burton et al. (2013) suggest that extraverted individuals are better at vocal emotion recognition overall, but the latter study only finds this effect for males. Moreover, Scherer and Scherer (2011) argued that neuroticism links to better overall recognition of vocal emotions, while Burton et al. (2013) and other studies failed to find this relationship (Cunningham, 1977; Terracciano et al., 2003; Banziger et al., 2009). Interestingly, a *negative* relationship was evident between openness to experience and recognition of positive emotions. Although this relationship is only evident for recognition of positive emotions specifically, this is still surprising considering that Terracciano et al. (2003) argued for a *positive* relationship between vocal emotion perception and openness to experience.

Overall, the present study did not confirm a pairwise linear relationship between overall emotion perception and specific personality traits, a finding supported by some previous studies (e.g., Cunningham, 1977; Banziger et al., 2009). However, it is still possible that individual differences in personality traits play a role in vocal emotion recognition; personality characteristics may influence *how quickly* rather than *how accurately* individuals process vocal emotions. Thus, Study 2 was designed to explore the temporal processing of vocal emotions and its potential relationship to personality traits.

## Study 2

Study 2 is the first attempt to explore whether individual differences in personality traits influence the time course processing of vocal emotions. Specifically, Study 2 aims to extend Study 1 by examining whether personality traits influence *how quickly*, in contrast to *how accurately*, different vocal emotion categories are identified. At a group level, we predicted that less acoustical information along the timeline would be required to accurately identify anger, fear, sadness, and neutral utterances compared to utterances intoned in a happy or disgusted voice, which would be in line with previous findings (e.g., Pell and Kotz, 2011). No clear predictions are made for the temporal unfolding of pleasant surprise, as this is, to our knowledge, the first study to examine this emotion category using a gating paradigm. Importantly, the study set out to examine the trait-congruency hypothesis; are extraverted and neurotic individuals *quicker* at recognizing positive and negative emotions, respectively.

### Methods

#### Participants

One hundred-and-one (86 females, mean age: 19.4, *SD*: 2.45) undergraduate Psychology students at the University of Essex participated as part of a module requirement and received credits in exchange for their participation. All participants gave their written informed consent and reported normal or corrected to normal hearing and vision. Comparable to Study 1, participants who gave a self-report that they were experiencing a mental health disorder were excluded from the analysis, resulting in 83 participants (64 females) included in the final analyses. Power analysis was conducted as for Study 1 with a sample of 83 being sufficient to detect a small to medium sized effect keeping these same criteria.

#### Materials

Semantically-anomalous pseudo-utterances (e.g., “Klaff the frisp dulked lantary,” see Appendix B for full list) spoken by a professional female actress were selected from a previous inventory (Paulmann and Uskul, 2014). In the original study, average accuracy rates for stimuli were much better than expected by chance (14.2%) ranging from 55% (for happiness) to 91% (for neutral). From this inventory, 14 utterances were selected, each one coming from one of the seven emotional categories (anger, disgust, fear, happy, neutral, pleasant surprise, sad). All utterances were seven syllables long and edited into six gate intervals using *Praat* (Boersma and Weenink, 2009) on a syllable by syllable basis with increasing duration (see Pell and Kotz, 2011, for a similar approach). Average gate duration was 260 ms, full sentences were on average 2.2 s long. The first gate spanned over two syllables while subsequent gates added one syllable each until the utterance was complete (6th gate). The same 14 utterances were presented in each of the six blocks, with increasing syllable length per block, and utterances were randomly allocated for each individual participant.

#### The Big Five Inventory

The BFI was used as measure to characterize individual personality traits, as described in Study 1.

#### Design

A cross-sectional design was used. For the correlational analyses, predictor variables were identical to Study 1 (i.e., personality traits) while the criterion variable was recognition accuracy (and confidence ratings) at each gate interval and identification point of the intended emotion (in ms). For the repeated-measures ANOVA, Emotion (seven levels; anger, disgust, fear, happy, neutral, pleasant surprise, and sad) and Gate (six levels; Gates 1 to 6) were treated as within-subject variables.

#### Procedure

The experimental procedure was identical to Study 1; however, participants now listened to segments of each gate or the complete utterance (in the last block) rather than only complete sentences. Also, they were asked to indicate how confident they were that they had identified the correct emotion after categorizing each stimulus. The confidence scale ranged from 1 (not confident at all) to 7 (very confident). The procedure employed was identical to the one employed in Pell and Kotz (2011).

#### Results

##### Vocal emotion recognition

Again, unbiased hit rates were calculated and arcsine-transformed to control for response biases (Wagner, 1993) (Appendix D tabulates raw hit rates together with error patterns of responding). Arcsine-transformed H_u_ scores and SDs for each emotion category at each gate interval are presented in Table 4.

**Table 4:** Study 2: mean arcsine-transformed H_u_ scores and SD for each emotion at each gate.

	**Gate identification**						
**Expression**	**Gate 1**	**Gate 2**	**Gate 3**	**Gate 4**	**Gate 5**	**Gate 6**	**Average**
Anger	0.75	0.90	0.96	1.00	1.05	1.11	0.96
SD	0.18	0.18	0.17	0.20	0.23	0.22	0.15
Disgust	0.37	0.48	0.57	0.64	0.68	0.80	0.59
SD	0.16	0.22	0.26	0.29	0.33	0.33	0.22
Fear	0.44	0.51	0.58	0.61	0.62	0.60	0.56
SD	0.17	0.20	0.19	0.23	0.23	0.24	0.16
Happy	0.23	0.27	0.33	0.35	0.38	0.39	0.32
SD	0.12	0.14	0.16	0.16	0.16	0.18	0.11
Neutral	0.54	0.59	0.71	0.74	0.77	0.75	0.68
SD	0.11	0.12	0.16	0.19	0.21	0.21	0.14
Pls.sur	0.49	0.48	0.52	0.54	0.54	0.54	0.52
SD	0.13	0.16	0.18	0.17	0.19	0.18	0.13
Sad	0.55	0.71	0.72	0.76	0.77	0.81	0.72
SD	0.16	0.21	0.21	0.23	0.26	0.24	0.18
Average	0.48	0.56	0.63	0.66	0.69	0.72	
SD	0.08	0.11	0.12	0.15	0.16	0.17	

*Gate 6 corresponds to a full utterance*.

A repeated-measures ANOVA was used to examine how vocal emotion recognition unfolds over time. Significance level was again adjusted using Keppel's rule (new *p* = 0.017) (Keppel, 1991) and the Greenhouse-Geisser correction was applied. A significant main effect was found for Emotion, *F*_(4.227, 346.654)_ = 242.097, *p* < 0.001, suggesting that emotion categories could be successfully distinguished from each other. *Post hoc* comparisons showed that all individual contrasts, except disgust and fear, and fear and pleasant surprise, are significantly different. As shown in Table 4, anger is again the most accurately recognized emotion while happy is the emotion category that is most poorly recognized. Additionally, a significant main effect of Gate was found, *F*_(3.451, 282.972)_ = 112.928, *p* < 0.001, suggesting that recognition accuracy differed across gates. *Post hoc* comparisons revealed that recognition accuracy were significantly different at all gate intervals. Table 4 lists the overall mean recognition accuracy at each gate, showing that participants got better at recognizing emotional tone of voice with each increasing gate.

A significant Gate by Emotion interaction was also found, *F*_(19.285, 1581.383)_ = 11.809, *p* < 0.001, indicating recognition differences across gates for the different emotion categories. The interaction was unfolded by emotion and *post hoc* comparisons revealed the following patterns: for angry stimuli, recognition rates improved with increasing gate duration (all *p*s < 0.001), except between Gates 3 and 4 (*p* = 0.068) and between Gates 5 and 6 (*p* = 0.025) where no significant improvements were observed. Looking at disgust stimuli, recognition rates improved significantly across gates except when comparing accuracy rates between Gate 4 and Gate 5 (*p* = 0.109). For stimuli expressing fear, recognition rates did not change significantly after listening to Gate 3 stimuli (all *p* ≥ 0.060), i.e., participants did not recognize fear better at longer durations. Comparable findings were observed for happy stimuli for which accuracy rates were not significantly different when comparing Gate 3 vs. Gate 4, Gate 4 vs. Gate 5, and Gate 5 vs. Gate 6 rates (all *p*s ≥ 0.02). Similarly, for neutral, recognition rates improved with increasing gate duration, except that recognition rates were not significantly different between at Gates 3 and 4, between Gates 4 and 5, and between Gates 5 and 6 (all *ps* ≥ 0.035). For pleasant surprise, recognition rates did not significantly improve on a gate by gate manner as contrast between Gates 1 and Gates 2, Gates 2 vs. 3, Gates 3 and 4, Gates 4 and 5, and between Gates 5 and 6 did not reach significance; still, at Gate 6, recognition was better than at Gate 1 (see Table 4), that is recognition improved with increasing exposure duration. Finally, for sadness, recognition improved with increasing stimulus duration, but comparisons for recognition rates between Gate 2 and Gate 3, Gates 3 and 4, Gates 4 and 5, and Gates 5 and 6 failed to reach significance (all *p*s ≥ 0.017). Overall, results showed that emotion recognition is generally easier when listening to longer vocal samples.

#### Vocal Emotion Processing and Personality

**Figure 2:**
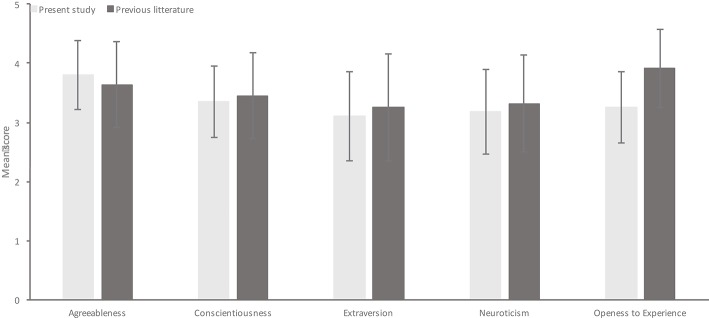
Means and SDs from the present study for each BFI variable including means and SDs obtained for the same variables in previous research. Means from the previous literature are based on results reported by Srivastava et al. (2003), where data from age group 20 was used, as in Study 1.

As for Study 1, means and SDs of all BFI variables were comparable to previous literature for general population samples (see Figure 2). Pearson's correlations were then conducted to examine the relationship between arcsine-transformed H_u_ scores and BFI variables at each gate interval. These results are presented in Table 5. While individuals scoring high on agreeableness and conscientiousness tended to have better overall recognition and recognition of negative emotions at Gate 6, extraverted individuals tended to have better recognition of positive emotions at this final gate. However, there are no clear and consistent trends between speed of recognition and BFI traits.

**Table 5:** Pearson's correlations (*r*-value) and their significance level (^*^p < 0.05, ^**^p < 0.01) between Hu scores at individual gates and the Big Five Inventory (BFI).

		**BFI variables**									
**Emotion recognition accuracy**		**Agreeableness**		**Conscientiousness**		**Extraversion**		**Neuroticism**		**Openness to experience**	
		***r*-value**	***p*-value**	***r*-value**	***p*-value**	***r*-value**	***p*-value**	***r*-value**	***p*-value**	***r*-value**	***p*-value**
Gate 1	Anger	0.036	0.750	−0.041	0.710	−0.022	0.846	0.295^**^	0.007	−0.038	0.735
	Disgust	−0.063	0.572	−0.019	0.862	0.152	0.171	−0.055	0.621	0.114	0.305
	Fear	0.088	0.430	0.060	0.592	0.015	0.891	−0.012	0.917	0.023	0.835
	Happy	−0.095	0.392	−0.038	0.733	0.058	0.604	0.040	0.718	0.176	0.112
	Neutral	0.042	0.705	0.097	0.383	0.204	0.064	0.178	0.108	0.059	0.599
	Pls.sur	0.106	0.342	−0.054	0.628	−0.016	0.885	0.156	0.160	0.059	0.597
	Sad	0.119	0.286	0.285^**^	0.009	0.133	0.232	0.067	0.544	0.041	0.715
	EmoAve	0.068	0.539	0.082	0.459	0.135	0.225	0.184	0.095	0.107	0.334
	AveNotNeu	0.066	0.556	0.068	0.539	0.101	0.363	0.162	0.143	0.105	0.345
	AveNegEm	0.072	0.517	0.108	0.330	0.107	0.338	0.130	0.243	0.053	0.637
	AvePosEm	0.011	0.923	−0.063	0.572	0.027	0.809	0.136	0.222	0.157	0.155
Gate 2	Anger	0.191	0.084	0.068	0.542	0.242^*^	0.027	0.012	0.912	0.041	0.716
	Disgust	0.036	0.746	0.044	0.692	0.220^*^	0.046	−0.043	0.700	0.180	0.103
	Fear	−0.037	0.738	0.050	0.656	0.133	0.232	0.021	0.849	0.031	0.782
	Happy	0.022	0.845	0.104	0.352	0.202	0.067	−0.139	0.210	−0.013	0.908
	Neutral	0.132	0.233	−0.009	0.935	0.203	0.065	0.026	0.816	0.055	0.621
	Pls.sur	−0.036	0.745	−0.077	0.487	−0.015	0.895	0.045	0.689	0.141	0.203
	Sad	0.088	0.429	0.108	0.331	0.030	0.787	0.172	0.121	−0.027	0.812
	EmoAve	0.084	0.448	0.069	0.533	0.220^*^	0.046	0.030	0.785	0.094	0.400
	AveNotNeu	0.070	0.528	0.078	0.484	0.207	0.060	0.029	0.795	0.093	0.401
	AveNegEm	0.091	0.411	0.093	0.404	0.213	0.054	0.056	0.614	0.081	0.469
	AvePosEm	−0.012	0.913	0.009	0.936	0.114	0.305	−0.054	0.629	0.092	0.409
Gate 3	Anger	0.122	0.272	0.016	0.884	0.068	0.543	0.106	0.341	0.122	0.273
	Disgust	0.116	0.296	0.156	0.160	0.130	0.243	−0.028	0.801	0.141	0.203
	Fear	0.084	0.450	0.069	0.534	0.119	0.284	−0.161	0.147	0.140	0.206
	Happy	0.052	0.642	0.040	0.719	0.164	0.138	0.000	0.997	−0.114	0.303
	Neutral	0.242^*^	0.027	0.203	0.066	0.260^*^	0.018	−0.118	0.288	−0.015	0.894
	Pls.sur	0.096	0.389	0.070	0.532	−0.014	0.902	0.099	0.376	0.076	0.494
	Sad	0.232^*^	0.034	0.103	0.355	0.065	0.558	0.034	0.762	0.023	0.840
	EmoAve	0.206	0.062	0.148	0.183	0.168	0.130	−0.015	0.892	0.094	0.400
	AveNotNeu	0.186	0.092	0.127	0.252	0.138	0.213	0.008	0.946	0.111	0.318
	AveNegEm	0.186	0.092	0.125	0.260	0.131	0.239	−0.019	0.862	0.143	0.198
	AvePosEm	0.094	0.399	0.070	0.532	0.087	0.435	0.066	0.556	−0.016	0.884
Gate 4	Anger	0.051	0.645	0.043	0.700	0.141	0.204	0.206	0.062	0.061	0.584
	Disgust	0.138	0.212	0.059	0.597	0.085	0.443	0.016	0.887	0.082	0.462
	Fear	0.069	0.535	0.109	0.327	0.121	0.276	0.008	0.942	0.106	0.342
	Happy	−0.054	0.627	0.112	0.314	0.060	0.592	0.031	0.778	0.025	0.821
	Neutral	0.115	0.300	0.128	0.250	0.170	0.126	−0.083	0.454	0.067	0.548
	Pls.sur	0.122	0.271	0.149	0.180	0.112	0.313	−0.001	0.990	0.168	0.130
	Sad	0.178	0.107	0.163	0.142	0.053	0.636	0.124	0.263	0.149	0.180
	EmoAve	0.135	0.224	0.149	0.180	0.147	0.184	0.063	0.572	0.133	0.229
	AveNotNeu	0.132	0.236	0.145	0.192	0.134	0.226	0.090	0.418	0.140	0.207
	AveNegEm	0.145	0.190	0.119	0.282	0.126	0.255	0.104	0.349	0.128	0.249
	AvePosEm	0.044	0.692	0.158	0.155	0.104	0.348	0.017	0.876	0.119	0.285
Gate 5	Anger	0.105	0.346	0.102	0.360	−0.004	0.971	0.137	0.216	0.050	0.651
	Disgust	0.092	0.408	0.106	0.342	0.017	0.879	0.084	0.451	0.077	0.490
	Fear	0.088	0.426	0.116	0.296	0.065	0.559	0.004	0.972	0.015	0.896
	Happy	0.037	0.742	−0.009	0.935	0.054	0.629	0.048	0.667	−0.079	0.478
	Neutral	0.069	0.535	0.137	0.215	0.199	0.071	0.070	0.529	0.056	0.617
	Pls.sur	0.096	0.387	0.083	0.456	−0.026	0.815	0.039	0.723	0.027	0.807
	Sad	0.208	0.059	0.134	0.226	0.115	0.303	0.126	0.257	−0.010	0.929
	EmoAve	0.146	0.189	0.141	0.202	0.082	0.462	0.106	0.339	0.036	0.745
	AveNotNeu	0.152	0.170	0.133	0.230	0.053	0.637	0.107	0.337	0.030	0.788
	AveNegEm	0.154	0.166	0.143	0.198	0.059	0.595	0.110	0.321	0.045	0.689
	AvePosEm	0.082	0.461	0.049	0.659	0.012	0.918	0.051	0.647	−0.024	0.829
Gate 6	Anger	0.077	0.489	0.146	0.188	0.127	0.254	0.136	0.220	0.083	0.454
	Disgust	0.140	0.205	0.165	0.135	0.032	0.776	0.149	0.179	0.177	0.110
	Fear	0.260^*^	0.018	0.167	0.132	0.193	0.080	0.053	0.632	0.129	0.244
	Happy	0.026	0.813	0.092	0.410	0.255^*^	0.020	−0.030	0.791	0.030	0.789
	Neutral	0.151	0.172	0.226^*^	0.040	0.211	0.055	0.056	0.614	0.100	0.369
	Pls.sur	0.119	0.283	0.179	0.105	0.168	0.130	−0.061	0.582	0.237^*^	0.031
	Sad	0.321^**^	0.003	0.269^*^	0.014	0.125	0.261	0.024	0.830	−0.039	0.729
	EmoAve	0.221^*^	0.045	0.244^*^	0.026	0.201	0.069	0.079	0.480	0.142	0.199
	AveNotNeu	0.225^*^	0.041	0.236^*^	0.032	0.189	0.087	0.079	0.475	0.144	0.193
	AveNegEm	0.248^*^	0.024	0.234^*^	0.033	0.141	0.204	0.119	0.282	0.120	0.278
	AvePosEm	0.086	0.441	0.160	0.150	0.249^*^	0.023	−0.054	0.631	0.157	0.157

*Abbreviations are identical to Table 3*.

Importantly, the emotion identification point (EIP) was calculated for each emotion category to establish how much acoustical information is needed for listeners to successfully identify the intended emotion category. For each participant, EIP was first calculated for each vocal stimulus and then averaged across each emotion category (see Jiang et al., 2015 for a similar calculation procedure). Further, EIP was averaged for each emotion category across participants. As seen Table 6, anger and disgust are the emotion categories recognized quickest and slowest, respectively.

**Table 6:** Identification points in milliseconds and SD for each emotion category and average identification point across all emotions.

	**Intended emotion**							
	**Anger**	**Disgust**	**Fear**	**Happy**	**Neutral**	**Pls.sur**	**Sad**	**Average**
Identification point (ms)	868	1526	1163	1263	888	1029	1035	1110
SD	222	332	258	246	271	228	278	262

A repeated-measures ANOVA was conducted to examine whether the emotion identification point (EIP) differed between the different emotion categories, and significance level was adjusted to 0.017 to correct for multiple comparisons (Keppel, 1991). A significant main effect was found for EIP, *F*_(5.318, 436.046)_ = 79.617, *p* < 0.001. *Post hoc* comparisons revealed that all EIPs were significantly different from each other for all emotion categories, except for contrasts between anger and neutral, and pleasant surprise and sadness. It is evident that anger (868 ms) and neutral (888 ms) are the emotion categories that are recognized quickest, while disgust (1,526 ms) is the emotion category that is recognized slowest.

Pearson's correlations were used to examine the relationship between EIPs and BFI variables. Again, no clear trends appeared between overall EIPs and any of the BFI measures (see Table 7). Further, confidence ratings (on a 1–7 point scale) and SD were calculated for each emotion category at each gate interval to assess how participants evaluated their own performance. Generally, confidence ratings increased as recognition accuracy increased, indicating that confidence judgments given by listeners are related to their actual vocal emotion recognition ability. However, confidence ratings were not related to personality traits (see Appendix E).

**Table 7:** Pearson's correlations (*R*-Value) and their significance level (^*^P < 0.05) between identification point in ms and the Big Five Inventory (BFI).

	**Identification point (MS) for each emotion**											
**Measure**		**Anger**	**Disgust**	**Fear**	**Happy**	**Neutral**	**Pls.sur**	**Sad**	**EmoAve**	**AveNotNeu**	**AveNegEm**	**AvePosEm**
Agreeableness	*r*-value	−0.020	−0.061	0.151	0.134	−0.109	0.033	−0.166	−0.020	0.009	−0.042	0.119
	*p*-value	0.857	0.586	0.174	0.229	0.325	0.766	0.133	0.856	0.934	0.705	0.283
Conscientiousness	*r*-value	−0.026	0.038	0.053	−0.084	−0.059	−0.045	−0.223^*^	−0.084	−0.077	−0.055	−0.091
	*p*-value	0.817	0.734	0.632	0.451	0.595	0.687	0.043	0.448	0.487	0.624	0.413
Extraversion	*r*-value	−0.036	−0.104	−0.062	−0.074	−0.203	0.042	−0.056	−0.132	−0.089	−0.098	−0.025
	*p*-value	0.748	0.348	0.578	0.508	0.066	0.707	0.617	0.236	0.426	0.379	0.820
Neuroticism	*r*-value	−0.202	0.121	0.057	−0.119	−0.075	−0.063	−0.215	−0.109	−0.100	−0.066	−0.129
	*p*-value	0.068	0.276	0.607	0.283	0.498	0.570	0.051	0.327	0.367	0.555	0.245
Openness to experience	*r*-value	0.021	−0.089	−0.101	0.034	−0.114	0.173	0.038	−0.022	0.008	−0.053	0.141
	*p*-value	0.849	0.425	0.365	0.758	0.307	0.119	0.732	0.842	0.941	0.634	0.204

*Abbreviations are identical to Table 3*.

#### Discussion

The overall aim of Study 2 was to explore whether individual differences in personality traits influenced *recognition speed* of vocal emotions. Firstly, group level analyses replicated findings in the previous vocal emotion literature. Specifically, as in previous studies, recognition accuracy improved at successive gate intervals and some emotion categories (i.e., anger and neutral) are recognized much quicker than other emotion categories (i.e., disgust and happiness; e.g., Cornew et al., 2010; Pell and Kotz, 2011; Rigoulot et al., 2013). Overall recognition accuracy patterns were also comparable with results obtained in Study 1 (e.g., anger and happiness were recognized with the highest and lowest accuracy respectively). Findings from both studies are thus well in line with the vocal emotion literature in general.

Secondly, in contrast to Study 1, extraverted individuals tended to be better at recognizing positive emotions at the final gate, while individuals scoring high on agreeableness and conscientiousness tended to be better at recognizing negative emotions and emotions overall at the final gate. It is unclear why we find these influences in Study 2, but did not observe them in Study 1. One crucial difference between the two studies is the stimuli used; in Study 2, we presented materials spoken by a professional speaker and that contained no semantic information. It could thus be argued that individual differences only appear when emotions are expressed in a highly prototypical way or when lacking semantic information. However, given that recognition rates across studies were comparable, speaker differences do not seem to influence emotion recognition accuracy heavily, it is thus less likely that the individual difference patterns are solely linked to the speaker differences across studies. Rather, if personality traits reliably influence the overall recognition ability of vocal emotions, this should have been evident in both Studies 1 and 2, which is not the case.

Importantly, the present study also failed to find a relationship between any of the personality traits and vocal emotion recognition *speed*. For instance, should the predictions from trait-congruency hypotheses be supported, a relationship should have been observed between extraversion and quicker EIPs for positive emotions, and for neuroticism and quicker EIPs for negative emotions. In short, no evidence was found here to support the assumption that personality traits heavily influence the temporal processing of vocal emotions.

## General Discussion

Based on the assumption that emotion processing is greatly influenced by personality characteristics (e.g., Davitz, 1964; Matsumoto et al., 2000; Hamann and Canli, 2004; Schirmer and Kotz, 2006), we designed two independent but related studies to explore how personality traits influence the *accuracy* and *speed* of vocal emotion recognition. We initially analyzed the data at a group level to ensure that the findings in both studies reflected the vocal emotion recognition literature in general.

### Vocal Emotion Processing and the Influence of Individual Personality Traits

The overall aim of the present investigation was to explore whether personality traits could explain variation in vocal emotion processing. In both studies, the data collected provided a solid base to explore this relationship; while the average scores on each personality dimension reflected a valid representation of general findings in the personality literature, the data on vocal emotion processing was also considered robust. While Study 1 reported an overall recognition accuracy of 55.3%, Study 2 reported an overall recognition accuracy of 61.8% at the final gate. While Study 1 presented materials from several untrained speakers, Study 2 employed materials from a professional female speaker. Thus, less speaker variability and potentially more prototypical portrayals of vocal emotions may have resulted in Study 2's higher average recognition accuracy. In both studies, recognition accuracy differed across emotions, in which anger and happy were the most accurately and most poorly recognized emotions, respectively. These results are in line with previous findings (e.g., Scherer, 1989; Paulmann et al., 2016).

In Study 2, the analyses of the time course processing of vocal emotions also showed that distinct emotion categories unfolded at different rates, suggesting that the amount of acoustical information required to identify the intended emotion differed between distinct emotion categories. These emotion-specific recognition patterns were consistent with the previous literature (e.g., Pell and Kotz, 2011). Also, recognition accuracy improved at successive gate intervals, in line with previous research (e.g., Cornew et al., 2010; Pell and Kotz, 2011; Rigoulot et al., 2013). One limitation of the gating design followed here is that segment duration increases over time (in order to determine the recognition point of individual emotions); however, this may limit our ability to compare the recognition success rates of short vs. long speech segments given that the longer segments were also heard *after* the short segments. To avoid this confound, future studies could randomly play short and long segments of speech to infer if longer gate durations indeed always lead to better recognition.

With regards to the relationship between vocal emotion processing and personality traits, we based our predictions on the trait-congruency hypothesis, which suggests that extraverted and neurotic individuals should display a bias toward processing of positive and negative emotions, respectively (e.g., Gomez et al., 2002; Robinson et al., 2007). It was not possible to formulate specific predictions for the other personality dimensions due to the sparse and contradictory findings in previous literature. We argued that, should a relationship between personality traits and vocal emotion processing be considered robust, findings for overall recognition accuracy would have to be replicated across the two studies.

Study 1 failed to support the predictions made by the trait-congruency hypotheses. Interestingly, the only personality trait that seemed to influence recognition accuracy of positive emotions was openness to experience. Specifically, individuals scoring lower on openness to experience were found to be better at recognizing positive vocal emotions. However, this result goes in the opposite direction to results reported previously (Terracciano et al., 2003), which suggest a positive relationship between openness to experience and recognition of vocal emotions.

Similarly, Study 2 also failed to find a significant relationship between personality traits and EIPs. Considering the adequate sample sizes, this suggests that individual variation in accuracy and speed of vocal emotion recognition cannot be clearly predicted by personality traits. A positive relationship was, however, found between extraversion and recognition of positive emotions at the final gate, suggesting that extraverted individuals are better at recognizing positive emotions overall. However, this finding was surprising, as Study 1 failed to find a relationship between extraversion and better recognition of positive emotions. Similarly, at Gate 6, agreeableness and conscientiousness were associated with better overall vocal emotion recognition and better recognition of negative emotions, but again, these findings are not reflected at different gates and are not consistent with results from EIPs or from results in Study 1.

Our findings are in line with previous studies that also failed to find a significant relationship between emotion perception and personality traits (e.g., Elfenbein et al., 2007; Banziger et al., 2009). Although there are more studies reporting a significant relationship (e.g., Cunningham, 1977; Scherer and Scherer, 2011; Burton et al., 2013) than studies reporting no relationship, it still raises the question of why replicating results is not guaranteed. One possibility, of course, is that samples are not comparable across studies. Here, we tried to address this concern by comparing the average scores for each personality dimension to general findings in the personality literature. We considered our average scores to be comparable. Additionally, it can be argued that observational study designs include only a restricted range of average scores (i.e., the scores that are most dense in the population, often mid-range scores). However, significant relationships may only be observed when including extremes from either end of the scale (which can easily be achieved in experimental designs). While this may be true, data from observational designs would still be valid with regard to their typicality in the population. That is, if a restricted range leads to non-significant findings while data including more extreme scores lead to a significant finding, the relationship between personality traits and vocal emotion recognition would still be overemphasized for the general population.

It is also worth noting that all studies that find a significant relationship between emotion recognition and vocal emotion perception do tend to provide an explanation for why this relationship is evident. For example, while Cunningham (1977) argues that neuroticism enhances emotion perception because discomfort is a motivating factor to perceive emotions. Scherer and Scherer (2011) who found the opposite pattern, argue that neurotic and anxious individuals might pay less attention to emotional cues from others. Thus, it seems easy to find plausible explanations, irrespective of the direction of the relationship. Future research should firstly focus on the discrepant results obtained in the personality and vocal emotion literature, and then try to gain a better understanding of the underlying reasons for the potential relationship(s).

If there is no clear and strong relationship between individual differences in personality traits and emotion processing, at least in the vocal domain, this can potentially explain why findings in the previous literature are so contradictory. We would argue that, as publishing null results is difficult, it is possible that at least some of the previous significant findings reflect chance findings. This hypothesis receives support from the fact that published studies showing null results are often reporting null findings in relation to other significant results. For example, the study by Elfenbein et al. (2007) focused mainly on the relationship between facial emotion recognition and effectiveness of negotiation, arguing that better facial emotion recognition could indeed influence negotiation performance. In relation to this, personality variables were also correlated against facial emotion recognition and null findings were reported as no relationships were found.

A limitation for the current investigation is the unequal male–female ratio in both Studies 1 and 2. Similar to other studies (e.g., Burton et al., 2013), our opportunity sampling resulted in a higher number of female participants. To address this limitation and to provide food for thought for future studies, we conducted *post-hoc* correlational analyses between personality traits and overall recognition accuracy for both studies for female and male participants separately. Similar to Burton et al. (2013) we fail to find reliable effects for our female sample. However, in latter study, the authors report a significant relationship between extraversion and conscientiousness and better vocal emotion recognition for male participants. Our current sample was too small to reliably comment on this relationship; yet, it may be of interest to some readers that we found a significant relationship between conscientiousness and overall emotion recognition (0.512; *p* = 0.021) in Study 1. No other effects were found in Study 1 or 2. Thus, it seems possible that previously reported significant associations between personality traits and emotion recognition (e.g., Terracciano et al., 2003; Scherer and Scherer, 2011) may predominantly have been driven by one gender only. Similarly, studies that fail to report significant associations might have overlooked relationships by collapsing across male and female participants. Thus, future studies with larger and more equal sample sizes should continue to explore how gender differences potentially influence the relationship between personality traits and vocal emotion processing. This will allow disentangling effects further and it is of great importance that future studies examine these points in a comprehensive and systematic manner. This will ensure that significant findings are replicable across different materials and different individuals when using same personality questionnaire measurements and research designs.

## Concluding Thoughts

These studies used sample sizes that were supported by power calculation as well as by previous studies that report relationships with even smaller samples (e.g., Scherer and Scherer, 2011; Burton et al., 2013). We also controlled for confounding variables by using the same measurement tool (i.e., BFI) consistently across both studies, and by exploring the effects of speaker variability and difference in sentence stimuli. Although the data on personality traits and vocal emotion processing was representative of findings in the personality and vocal emotion recognition literature in general, a pairwise linear relationship between personality traits and emotion categories was not identified. Taken together, these data allow predicting that an overemphasis on the role of personality on vocal emotion processing has been put forward in the past. Crucially, it seems as if relationships between individual differences and emotional tone of voice are more complex than previously assumed. We thus encourage future studies to explore this complex relationship in more detail to shed further light on this issue.

## Ethics Statement

This study was carried out in accordance with the recommendations of the University of Essex Science and Health Faculty Ethics Sub-committee. All subjects gave written informed consent in accordance with the Declaration of Helsinki. The protocol was approved by the University of Essex Science and Health Faculty Ethics Sub-committee.

## Author Contributions

DF worked on data collection, data analysis, and prepared draft of manuscript. HB worked on data analysis and manuscript draft. RM worked on manuscript draft. SP designed and programmed experiments, overlooked project, worked on data analysis and manuscript.

### Conflict of Interest Statement

The authors declare that the research was conducted in the absence of any commercial or financial relationships that could be construed as a potential conflict of interest.

## References

[B1] Airas M. Alku P. (2006). Emotions in vowel segments of continuous speech: analysis of the glottal flow using the normalised amplitude quotient. Phonetica 63, 26–46. 10.1159/000091405 16514274

[B2] Banse R. Scherer K. R. (1996). Acoustic profiles in vocal emotion expression. J. Pers. Soc. Psychol. 70, 614–636. 10.1037/0022-3514.70.3.614 8851745

[B3] Banziger T. Grandjean D. Scherer K. R. (2009). Emotion recognition from expressions in face, voice, and body: the Multimodal Emotion Recognition Test (MERT). Emotion 9, 691–704. 10.1037/a0017088 19803591

[B4] Banziger T. Scherer K. R. (2005). The role of intonation in emotional expressions. Speech Commun. 46, 252–267. 10.1016/j.specom.2005.02.016

[B5] Boersma P. Weenink D. (2009). Praat: doing phonetics by computer [Computer program]. Version 5.1.25. Available online at: http://www.praat.org/

[B6] Burton L. Bensimon E. Allimant J. M. Kinsman R. Levin A. Kovacs L. (2013). Relationship of prosody perception to personality and aggression. Curr. Psychol. 32, 275–280. 10.1007/s12144-013-9181-6

[B7] Cornew L. Carver L. Love T. (2010). There's more to emotion than meets the eye: a processing bias for neutral content in the domain of emotional prosody. Cogn. Emot. 24, 1133–1152. 10.1080/02699930903247492 21552425 PMC3088090

[B8] Costa Jr P. T. McCrae R. R. (1995). Domains and facets: hierarchical personality assessment using the revised NEO personality inventory. J. Pers. Assess. 64, 21–50. 10.1207/s15327752jpa6401_2 16367732

[B9] Costa P. McCrae R. (1989). Neo Five-Factor Inventory (NEO-FFI). Odessa, FL: Psychological Assessment Resources.

[B10] Costa P. T. McCrae R. R. (1992). Normal personality assessment in clinical practice: the NEO personality inventory. Psychol. Assess. 4, 5–13. 10.1037/1040-3590.4.1.5

[B11] Cunningham R. M. (1977). Personality and the structure of the nonverbal communication of emotion. J. Person. 45, 564–584. 10.1111/j.1467-6494.1977.tb00172.x 592086

[B12] Davitz J. R. (1964). The Communication of Emotional Meaning. New York, NY: McGraw-Hill.

[B13] Elfenbein H. A. Foo M. D. White J. Tan H. H. Aik V. C. (2007). Reading your counterpart: the benefit of emotion recognition accuracy for effectiveness in negotiation. J. Nonverbal Behav. 31, 205–223. 10.1007/s10919-007-0033-7

[B14] Faul F. Erdfelder E. Lang A. G. Buchner A. (2007). G^*^Power 3: a flexible statistical power analysis program for the social, behavioral, and biomedical sciences. Behav. Res. Methods 39, 175–191. 10.3758/BF03193146 17695343

[B15] Goldberg L. R. (1992). The development of markers for the big-five factor structure. Psychol. Assess. 4, 26–42. 10.1037/1040-3590.4.1.26

[B16] Gomez R. Gomez A. Cooper A. (2002). Neuroticism and extraversion as predictors of negative and positive emotional information processing: comparing Eysenck's, Gray's, and Newman's theories. Eur. J. Personal. 16, 333–350. 10.1002/per.459

[B17] Graham C. R. Hamblin A. W. Feldstain S. (2001). Recognition of emotion in English voices by speakers of Japanese, Spanish and English. Int. Rev. Appl. Ling. Lang. Teach. 39, 19–37. 10.1515/iral.39.1.19

[B18] Grosjean F. (1996). Gating. Lang. Cogn. Process. 11, 597–604. 10.1080/016909696386999

[B19] Hamann S. Canli T. (2004). Individual differences in emotion processing. Curr. Opin. Neurobiol. 14, 233–238. 10.1016/j.conb.2004.03.010 15082330

[B20] Jiang X. Paulmann S. Robin J. Pell M. D. (2015). More than accuracy: nonverbal dialects modulate the time course of vocal emotion recognition across cultures. J. Exp. Psychol. Hum. Percept. Perform 41, 597–612. 10.1037/xhp0000043 25775176

[B21] John O. P. Donahue E. M. Kentle R. L. (1991). The Big Five Inventory–Versions 4a and 54. Berkley, CA: University of California, Berkeley; Institute of Personality and Social Research.

[B22] John O. P. Neumann L. P. Soto C. J. (2008). Paradigm shift to the integrative big-five trait taxonomy: history, measurement, and conceptual issues, in Handbook of Personality: Theory and Research, eds John O. P. Robins R. W. Pervin L. A. (New York, NY: Guilford Press), 114–158.

[B23] Juslin P. N. Laukka P. (2001). Impact of intended emotion intensity on cue utilization and decoding accuracy in vocal expression of emotion. Emotion 1, 381–412. 10.1037/1528-3542.1.4.381 12901399

[B24] Keppel G. (1991). Design and Analysis: A Researcher's Handbook. Prentice-Hall, Inc.

[B25] Kohler C. G. Turner T. H. Bilker W. B. Brensinger C. M. Siegel S. J. Kanes S. J.. (2003). Facial emotion recognition in schizophrenia: intensity effects and error pattern. Am. J. Psychiatry 160, 1768–1774. 10.1176/appi.ajp.160.10.1768 14514489

[B26] Larsen R. J. Ketelaar T. (1989). Extraversion, neuroticism and susceptibility to positive and negative mood induction procedures. Person. Individ. Diff. 10, 1221–1228. 10.1016/0191-8869(89)90233-X

[B27] Leppanen J. M. Milders M. Bell J. S. Terriere E. Hietanen J. K. (2004). Depression biases the recognition of emotionally neutral faces. Psychiatry Res. 128, 123–133. 10.1016/j.psychres.2004.05.020 15488955

[B28] Matsumoto D. LeRoux J. Wilson-Cohn C. Raroque J. Kooken K. Ekman P. (2000). A new test to measure emotion recognition ability: matsumoto and ekman's Japanese and Caucasian brief affect recognition test (JACBART). J. Nonverbal Behav. 24, 179–209. 10.1023/A:1006668120583

[B29] Paulmann S. Furnes D. Bokenes A. M. Cozzolino P. J. (2016). How psychological stress affects emotional prosody. PLoS ONE 11:e0165022. 10.1371/journal.pone.0165022 27802287 PMC5089770

[B30] Paulmann S. Uskul A. K. (2014). Cross-cultural emotional prosody recognition: evidence from Chinese and British listeners. Cogn. Emot. 28, 230–244. 10.1080/02699931.2013.812033 23862740

[B31] Pell M. D. Kotz S. A. (2011). On the time course of vocal emotion recognition. PLoS ONE 6:e27256. 10.1371/journal.pone.0027256 22087275 PMC3210149

[B32] Rigoulot S. Wassiliwizky E. Pell M. D. (2013). Feeling backwards? How temporal order in speech affects the time course of vocal emotion recognition. Front. Psychol. 4:367. 10.3389/fpsyg.2013.00367 23805115 PMC3690349

[B33] Robinson M. D. Ode S. Moeller S. K. Goetz P. W. (2007). Neuroticism and affective priming: evidence for a neuroticism-linked negative schema. Pers. Individ. Dif. 42, 1221–1231. 10.1016/j.paid.2006.09.027 18449325 PMC1892199

[B34] Rubin R. S. Muntz D. C. Bommer W. H. (2005). Leading from within: the effects of emotion recognition and personality on transformational leadership behavior. Acad. Manag. J. 48, 845–858. 10.5465/amj.2005.18803926

[B35] Rusting C. L. (1998). Personality, mood, and cognitive processing of emotional information: three conceptual frameworks. Psychol. Bull. 124, 165–196. 10.1037/0033-2909.124.2.165 9747185

[B36] Scherer K. R. (1989). Emotion psychology can contribute to psychiatric work on affect disorders: a review. J. R. Soc. Med. 82, 545–547. 10.1177/014107688908200913 2677371 PMC1292302

[B37] Scherer K. R. (1995). Expression of emotion in voice and music. J. Voice 9, 235–248. 10.1016/S0892-1997(05)80231-0 8541967

[B38] Scherer K. R. Scherer U. (2011). Assessing the ability to recognize facial and vocal expressions of emotion: construction and validation of the emotion recognition index. J. Nonverbal Behav. 35, 305–326. 10.1007/s10919-011-0115-4

[B39] Schirmer A. Kotz S. A. (2006). Beyond the right hemisphere: brain mechanisms mediating vocal emotional processing. Trends Cogn. Sci. 10, 24–30. 10.1016/j.tics.2005.11.009 16321562

[B40] Srivastava S. John O. P. Gosling S. D. Potter J. (2003). Development of personality in early and middle adulthood: set like plaster or persistent change? J. Personal. Soc. Psychol. 84:1041 10.1037/0022-3514.84.5.1041 12757147

[B41] Terracciano A. Merritt M. Zonderman A. B. Evans M. K. (2003). Personality traits and sex differences in emotion recognition among African Americans and Caucasians. Ann. N.Y. Acad. Sci. 1000, 309–312. 10.1196/annals.1280.032 14766644 PMC2580736

[B42] Toivanen J. Waaramaa T. Alku P. Laukkanen A. M. Seppanen T. Vayrynen E. (2006). Emotions in [a]: a perceptual and acoustic study. Logoped Phoniatr. Vocol. 31, 43–48. 10.1080/14015430500293926 16517522

[B43] Unoka Z. Fogd D. Fuzy M. Csukly G. (2011). Misreading the facial signs: specific impairments and error patterns in recognition of facial emotions with negative valence in borderline personality disorder. Psychiatry Res. 189, 419–425. 10.1016/j.psychres.2011.02.010 21429593

[B44] Wagner H. L. (1993). On measuring performance in category judgment studies of nonverbal behavior. J. Nonverbal Behav. 17, 3–28. 10.1007/BF00987006

[B45] Williams C. E. Stevens K. N. (1972). Emotions and speech: some acoustical correlates. J. Acoust. Soc. Am. 52, 1238–1250. 10.1121/1.1913238 4638039

