# Supplementary material for: Exploring the Effects of Personality Traits on the Perception of Emotions From Prosody
Source: Front Psychol. 2019 Feb 12;10:184. doi: 10.3389/fpsyg.2019.00184 (PMC6385770; doi:10.3389/fpsyg.2019.00184)
Supplement: Supplementary file 1 [file Data_Sheet_1.docx]

Appendix A

The 15 sentences uttered by the speakers

1. The fence was painted brown.
2. The dog had two owners.
3. The book was green.
4. It was a heavy car.
5. The cat has night vision.
6. The water bottle was full.
7. The shop sells many things.
8. The boxes contained many items.
9. The horse was eating an apple.
10. The bird flew over the house.
11. There was food in the fridge.
12. This is a yellow blanket.
13. The top was made of cotton.
14. The woman crossed the street.
15. The man posted a card.

Appendix B

The 14 pseudo-utterances portrayed by the female actress

1. Klaff the frisp dulked lantary
2. Ganted the crasp blart fasket
3. Flob hobbered the foler frall
4. Controft jankus the curlod
5. Vian lorb dolan the wance
6. Janded the rendered hindum
7. Homit the gattast thintle
8. Spinst the ronsent doop dant
9. Humla dwarrd the ivwa crot
10. Posal windered the pample
11. Chinter fintest the romal
12. Mooled chumpet the zilted
13. Entrine the zoomit bandoom
14. Tantalint plad the bunner

Appendix C

Table C1

*Average Recognition Accuracy in Percentage for Each Emotional*
*Category and Confusion Patterns of Error Responding (Study 1)*.

| Response Given | | | | | | | |
| --- | --- | --- | --- | --- | --- | --- | --- |
| Expression | Anger | Disgust | Fear | Happy | Neutral | Pls. Sur | Sad |
| Anger | 66.1% | 13.9% | 1.7% | 1.5% | 10.1% | 5.3% | 1.0% |
| Disgust | 6.4% | 42.6% | 2.1% | 5.9% | 18.9% | 16.7% | 6.9% |
| Fear | 6.1% | 4.0% | 35.2% | 4.8% | 12.7% | 11.3% | 25.4% |
| Happy | 1.6% | 1.0% | 1.3% | 33.8% | 41.0% | 19.4% | 1.5% |
| Neutral | 2.1% | 2.7% | 1.4% | 1.8% | 67.3% | 1.4% | 22.9% |
| Pls. Sur | 1.2% | 2.4% | 2.1% | 15.8% | 3.0% | 74.9% | 0.3% |
| Sad | 1.5% | 4.9% | 4.1% | 0.7% | 20.4% | 1.0% | 67.0% |

*Note.* Missing percentages to 100% is due to invalid responses. Pls.sur = pleasant surprise.

Appendix D

*Note.* Gate 6 corresponds to a full utterance. Missing percentages to 100% is due to invalid responses.

Table D1

*Error Patterns for Each Emotion at Each Gate Interval (Study 2).*

Appendix E

Confidence ratings

Confidence ratings (on a 1-7 point scale) and SD were calculated for each emotion category at each gate interval. Further, confidence scores and SD were then calculated for each emotion category across gates and for each gate across emotions. These data are presented in Table E1.

As can be seen in Table E1, anger is the emotion category that achieved the highest confidence score overall, followed by sadness. The lowest confidence ratings are given for happy utterances. At Gate 1, sad and anger receives the highest confidence ratings while the lowest confidence ratings are given to happy and disgust utterances. Further, anger, disgust, and sad achieves the highest confidence score at Gate 6, while fear and happy receives the lowest confidence ratings.

Table E1

*Average confidence score and SD at each gate interval*

These patterns of confidence ratings are comparable to accuracy scores, indicating that confidence judgments given by listeners are related to their actual vocal emotion recognition ability.

Pearson’s correlations were conducted to examine the relationship between average confidence ratings at each gate and individual difference variables. Table E2 lists the correlations between confidence ratings at each gate and BFI. As can be seen from the table, no significant correlations were obtained.

Further, Pearson’s correlations were also conducted to examine the relationship between average confidence ratings for each emotion category across gates and individual difference variables. Table E3 lists correlations between confidence ratings for each emotion category across gates and BFI. No significant correlations were obtained.

Table E2

*Pearson’s correlations (r-value) and their significance level between confidence ratings at each gate interval and the BFI.*

| Average confidence ratings at each gate | | | | | | | | |
| --- | --- | --- | --- | --- | --- | --- | --- | --- |
| Measure |  | Gate 1 | Gate 2 | Gate 3 | Gate 4 | Gate 5 | Gate 6 | Average |
| Agreeableness | r-value  p-value | 0.043  0.699 | -0.05  0.653 | -0.029  0.792 | -0.036  0.749 | -.007  0.947 | -0.079  0.48 | -0.026  0.720 |
| Conscientiousness | r-value  p-value | 0.117  0.293 | 0.084  0.451 | 0.051  0.647 | 0.074  0.507 | 0.086  0.44 | 0.094  0.397 | 0.084  0.456 |
| Extraversion | r-value  p-value | 0.08  0.475 | 0.065  0.559 | 0.014  0.901 | 0.011  0.921 | -0.022  0.844 | 0.018  0.869 | 0.028  0.762 |
| Neuroticism | r-value  p-value | -0.092  0.409 | -0.027  0.81 | -0.003  0.98 | -0.015  0.895 | 0.043  0.701 | 0.024  0.833 | -0.012  0.771 |
| Openness to Experience | r-value  p-value | -0.016  0.885 | 0.07  0.528 | 0.024  0.831 | 0.092  0.407 | 0.145  0.19 | 0.169  0.128 | 0.081  0.495 |

Table E3

*Study 2: Pearson’s correlations (r-value) and their significance level between confidence ratings for each emotion category averaged across gates and the BFI.*

| Confidence rating of each emotion across gates | | | | | | | | |  |  |  |  |
| --- | --- | --- | --- | --- | --- | --- | --- | --- | --- | --- | --- | --- |
| Measure |  | Anger | Dis-gust | Fear | Happy | Neu-tral | Pls.Sur | Sad | Emo-Ave | Ave-NotNeu | Ave-NegEmo | Ave-PosEm |
| Agreeableness | r-value  p-value | 0.004  0.974 | -0.021  0.85 | -0.07  0.532 | -0.062  0.581 | -0.065  0.562 | 0.042  0.706 | -0.018  0.872 | -0.027  0.725 | -0.033  0.762 | -0.026  0.807 | -0.010  0.644 |
| Conscientiousness | r-value  p-value | 0.089  0.421 | 0.066  0.554 | 0.089  0.425 | 0.052  0.639 | 0.056  0.614 | 0.132  0.234 | 0.106  0.339 | 0.084  0.461 | 0.080  0.476 | 0.088  0.435 | 0.092  0.437 |
| Extraversion | r-value  p-value | -0.007  0.947 | 0.061  0.583 | -0.039  0.728 | 0.037  0.738 | 0.072  0.52 | 0.016  0.886 | 0.044  0.695 | 0.026  0.728 | 0.019  0.738 | 0.015  0.738 | 0.027  0.812 |
| Neuroticism | r-value  p-value | 0.047  0.676 | -0.067  0.548 | -0.003  0.978 | -0.023  0.84 | -0.034  0.764 | -0.007  0.951 | 0.02  0.859 | -0.010  0.802 | -0.005  0.780 | -0.001  0.765 | -0.015  0.896 |
| Openness to Experience | r-value  p-value | 0.039  0.728 | 0.091  0.412 | 0.028  0.8 | 0.129  0.246 | 0.143  0.199 | 0.064  0.565 | 0.075  0.503 | 0.081  0.493 | 0.072  0.538 | 0.058  0.611 | 0.097  0.406 |

*Note: Abbreviations are identical to Table 3.*
